# Risk Factors for Mortality Among Patients with Bloodstream Infection Caused by *Candidozyma auris* in Colombia

**DOI:** 10.3390/antibiotics15080758

**Published:** 2026-08-07

**Authors:** Mariana Pimiento-Díaz, Cynthia Ortiz-Roa, Sebastián Felipe Sierra-Umaña, José Yesid Rodríguez, Gerardo Antonio Muñetón-López, Carlos Augusto Solórzano-Ramos, Patricia Escandón, Carlos Arturo Álvarez-Moreno, Jorge Alberto Cortés

**Affiliations:** 1 Department of Internal Medicine, Faculty of Medicine, Universidad Nacional de Colombia, Sede Bogotá 111321, Colombia; mpimiento@unal.edu.co (M.P.-D.); caalvarezmo@unal.edu.co (C.A.Á.-M.); 2 Subred Integrada de Servicios de Salud Norte E.S.E. Hospital Simón Bolívar, Bogotá 111221, Colombia; cortizro@unal.edu.co; 3 Subred Integrada de Servicios de Salud Sur E.S.E. Hospital Tunal, Bogotá 110621, Colombia; sfsierrau@unal.edu.co; 4 Clínica Integral de Emergencias Laura Daniela, Facultad de Ciencias Médicas y de la Salud, Instituto Cardiovascular del Cesar, Centro de Investigaciones Microbiológicas del Cesar (CIMCE), Universidad de Santander, Valledupar 200001, Colombia; jos.rodriguez@mail.udes.edu.co; 5 Subred Integrada de Servicios de Salud Suroccidente E.S.E. Hospital de Kennedy, Bogotá 110871, Colombia; gamunetonl@unal.edu.co (G.A.M.-L.); csolorzanor@unal.edu.co (C.A.S.-R.); 6 Grupo de Microbiología, Instituto Nacional de Salud, Bogotá 111321, Colombia; pescandon@ins.gov.co; 7 Clínica Universitaria Colombia, Clínica Colsanitas, Bogotá 111321, Colombia; 8 Infectious Diseases Unit, Hospital Universitario Nacional, Bogootá 111321, Colombia

**Keywords:** *Candida auris*, *Candida*, mortality, risk factors, candidemia, Colombia

## Abstract

**Background**: *Candidozyma auris* (*Candida auris*) is an emerging multidrug-resistant fungal pathogen that can cause hospital outbreaks and is associated with high mortality. However, data on the clinical predictors of mortality in patients with bloodstream infection (BSI) caused by *C. auris* remain limited, particularly in Latin America, and the impact of guideline-based management practices on clinical outcomes has been scarcely explored. **Methods**: We conducted a multicenter retrospective cohort study including 134 patients with culture-proven *C. auris* BSI admitted to seven hospitals in Colombia between 2016 and 2021. Demographic, clinical, and therapeutic variables were evaluated to identify factors associated with 30-day mortality using a multivariate logistic regression model. Variables related to quality of care and guideline-based candidemia management, included in the EQUAL Candida Score, were also assessed. **Results**: The thirty-day mortality rate was 38.1%. In the multivariate analysis, older age (OR per decade: 1.37; 95% CI: 1.07–1.78), prior corticosteroid therapy (OR: 4.06; 95% CI: 1.70–10.16), and shock within 48 h of BSI (OR: 6.22; 95% CI: 2.52–16.52) were associated with higher mortality. Conversely, a higher EQUAL Candida Score was associated with lower 30-day mortality (OR: 0.84; 95% CI: 0.73–0.97). **Conclusions**: In this large Colombian cohort, advanced age, corticosteroid exposure, and early hemodynamic instability were significant predictors of mortality in patients with *C. auris* BSI. Greater adherence to recommended management practices—as reflected by the EQUAL Candida Score—appeared to be associated with lower mortality, underscoring the potential role of timely, structured care strategies in improving outcomes for patients with *C. auris* infections. To the best of our knowledge, this study is the first to evaluate the EQUAL Candida Score in a cohort of patients with *C. auris* BSI.

## 1. Introduction

Since its identification in 2009, *Candidozyma auris* (*Candida auris*) has emerged as a high-impact fungal pathogen in healthcare settings and has become a major global public health concern due to its resistance to multiple classes of antifungal agents and its ability to adhere to and colonize human skin and contaminate environmental surfaces [1]. These characteristics enable its persistence in hospital environments and facilitate the development of invasive infections in vulnerable hosts. The World Health Organization (WHO) has designated *C. auris* as a critical-priority fungal pathogen owing to its clinical and epidemiological significance, particularly among critically ill patients [2]. In Colombia, *C. auris* has been responsible for multiple hospital outbreaks since its initial isolation in 2015. The subsequent publication of the National Epidemiological Alert highlighted its growing public health importance and the substantial disease burden in intensive care units and among patients with severe comorbidities [3,4,5,6]. This situation is further complicated by diagnostic challenges, as *C. auris* may be misidentified by conventional phenotypic and biochemical methods in settings where MALDI-TOF MS or molecular methods are not routinely available, thereby hampering timely detection and facilitating its silent dissemination [7].

*Candidozyma auris* has become an important cause of bloodstream infection (BSI) in healthcare settings worldwide, particularly in intensive care units, where candidemia is among the most common invasive fungal infections. In this context, up to 25% of previously colonized patients may develop *C. auris* BSI. In a 2014 study from India, *C. auris* accounted for approximately 5.2% of candidemia cases, whereas reports from selected hospitals in Colombia have documented proportions as high as 26% [4,8,9]. These invasive infections, particularly those caused by multidrug-resistant (MDR) strains, are associated with high case fatality rates, ranging from 33% to 72% in international cohorts [10]. In Colombia, the crude mortality rate for *C. auris* BSI has approached 40% in some cohorts [4]. In addition, *C. auris* is characterized by a distinctive antifungal susceptibility profile, including high rates of fluconazole resistance, variable susceptibility to amphotericin B, and generally preserved activity of echinocandins. Antifungal susceptibility patterns vary considerably across geographic regions, largely reflecting the distribution of distinct phylogenetic clades [3,10,11].

Despite the growing number of reports describing the epidemiology of *C. auris* BSI, data on predictors of mortality remain limited and heterogeneous, particularly in middle-income countries, where the burden of disease is greatest. Most available studies are limited by small sample sizes, single-center designs, or the lack of standardized assessments of quality of care [12]. Recent multicenter cohorts have expanded the clinical characterization of *C. auris* bloodstream infection. However, most focus primarily on clinical and microbiological outcomes or compare prognosis with that of candidemia caused by other *Candida* species rather than integrating prognostic factors with structured measures of quality of care [4,13,14,15].

The EQUAL Candida Score, developed by the European Confederation of Medical Mycology (ECMM) based on the strongest recommendations for the diagnosis and management of candidemia in current guidelines, is a tool for assessing guideline adherence in clinical practice [13]. It incorporates key components of evidence-based care—including adequate blood culture sampling, species identification with subsequent susceptibility testing, empirical treatment with echinocandins and de-escalation to fluconazole when appropriate, routine echocardiography and ophthalmoscopy to evaluate deep-seated infection, follow-up blood cultures, and the recommended duration of antifungal therapy—thereby providing a structured framework for evaluating quality of care. The EQUAL Candida Score has previously been validated in patients with *Candida tropicalis* candidemia in a retrospective cohort study conducted in Thailand, in which higher scores were significantly associated with lower mortality in a multivariate regression model [14]. However, to the best of our knowledge, its performance in cohorts of patients with *C. auris* bloodstream infection has not been specifically evaluated.

In this context, we conducted a multicenter retrospective cohort study in Colombia to identify host-, disease-, and care-related factors associated with 30-day mortality in patients with *C. auris* bloodstream infection, with particular emphasis on the contribution of quality-of-care indicators as measured by the EQUAL Candida Score.

## 2. Results

**Figure 1:**
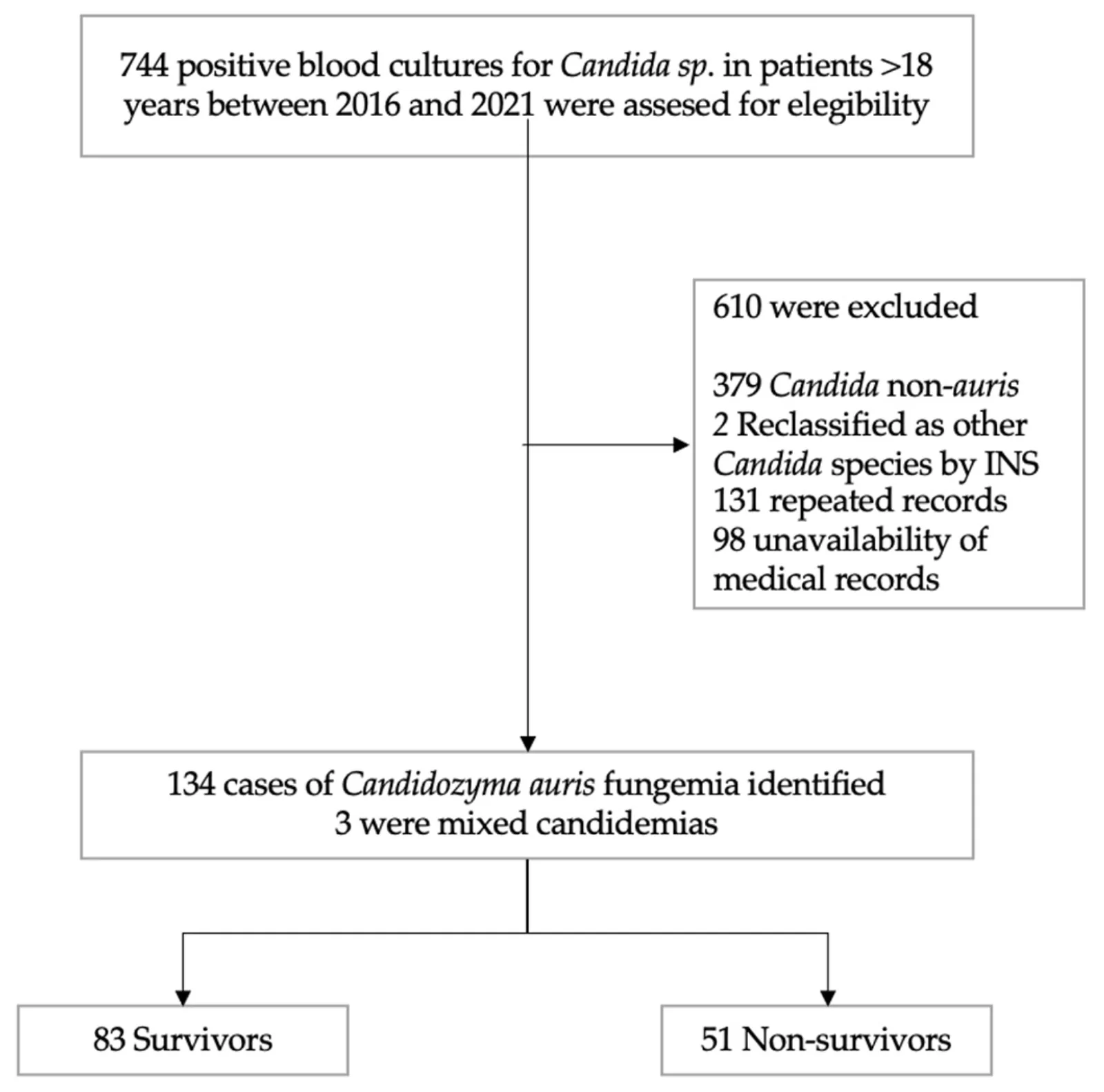
Flowchart of retrospective cohort of Colombian patients from seven tertiary care hospitals in Colombia, 2016–2021.

A retrospective cohort of 134 patients with culture-confirmed *C. auris* bloodstream infection was assembled from seven tertiary care hospitals in Colombia between 2016 and 2021, as illustrated in Figure 1. The baseline clinical and demographic characteristics of the cohort are presented in Table 1. The mean age was 54.2 years (SD 17.1), and two-thirds of the patients were men (64.2%). COVID-19 was the most common underlying condition (35.1%), followed by cardiovascular disease (32.8%) and diabetes mellitus (22.4%).

With respect to predisposing factors, 86.6% of patients were admitted to the intensive care unit (ICU), 61.9% required mechanical ventilation, 24.6% received parenteral nutrition, and 13.4% underwent renal replacement therapy. Corticosteroid therapy was administered to 40 patients (29.9%) and was significantly associated with COVID-19 (32/47 [68.1%]; *p* < 0.001).

Antifungal susceptibility testing for fluconazole and echinocandins (caspofungin) was performed for 51 isolates (38%). Resistance to fluconazole was observed in 38 isolates (74.5%), whereas all 51 isolates (100%) remained susceptible to echinocandins (caspofungin). Susceptibility testing for amphotericin B was not available.

### Treatment and Survival

Management variables are presented in Table 2. Overall, 119 patients (88.8%) received systemic antifungal therapy, but only 48 (35.8%) received timely treatment. Central venous catheter (CVC) removal was performed in 86 patients (64.2%), with similar proportions among survivors (66.3%) and non-survivors (60.8%; *p* = 0.648). Follow-up blood cultures were obtained from 83 patients (61.9%), but only 3 (3.6%) had cultures drawn within the first 72 h of diagnosis.

Overall, 66 patients (49.3%) died during follow-up, with most deaths (51 cases, 38.1%) occurring within the first 30 days. Late mortality (>30 days) accounted for 15 deaths (22.7% of all deaths). Table 1 displays the characteristics of survivors and non-survivors at 30 days after bloodstream infection. Patients who died were, on average, older than those who survived (59.4 vs. 51.1 years; *p* = 0.005). Although no significant differences were observed in sex or most comorbidities, chronic heart failure (11.8% vs. 1.2%; *p* = 0.023) and COVID-19 (45.1% vs. 28.9; *p* = 0.086) were more common among non-survivors. Predisposing clinical factors, including ICU admission, mechanical ventilation, and parenteral nutrition, did not differ significantly between survivors and non-survivors.

In the multivariate analysis, three factors were independently associated with increased 30-day mortality (Table 3): older age (per decade; OR 1.37, 95% CI: 1.07–1.78), previous corticosteroid use (OR 4.06, 95% CI: 1.70–10.16), and shock within 48 h of BSI (OR 6.22, 95% CI: 2.52–16.52). In contrast, a higher EQUAL Candida Score was associated with lower 30-day mortality (OR 0.84, 95% CI: 0.73–0.97). After multivariate analysis, no relationship was found between COVID-19 and mortality, despite the observed correlation between COVID-19 and previous corticosteroid use. The final model was evaluated for collinearity (none detected), goodness of fit (Hosmer–Lemeshow test = 0.33), and discrimination (area under the receiver operating characteristic curve (AUC-ROC) = 0.80).

## 3. Discussion

This retrospective multicenter cohort study, the largest in Latin America to date, provides a detailed characterization of patients with *C. auris* bloodstream infection treated in tertiary hospitals across Colombia. The study population reflects the high-risk profile typically associated with invasive fungal infections, including a predominance of critically ill patients, high rates of ICU admission, and multiple comorbidities. We found three factors associated with mortality: age, previous corticosteroid use, and shock within 48 h of BSI, while a higher EQUAL Candida Score was related to lower mortality. The 30-day mortality rate found (38.1%) is consistent with prior reports from both high- and middle-income settings, emphasizing the aggressive nature of *C. auris* infection [12,15].

Age was found to be an independent predictor of higher risk of 30-day mortality (odds ratio of 1.37 per decade, 95% CI: 1.07–1.78). Although data specific to age as a prognostic factor in *C. auris* BSI remain scarce, this finding aligns with prior studies identifying advanced age as an independent predictor of risk of *C. auris* infection and a determinant of poor prognosis in candidemia caused by other *Candida* species, likely reflecting a diminished physiological reserve and greater burden of comorbidities [15]. A Colombian multicenter cohort study of 109 patients reported a comparable per-decade increase in risk of mortality (HR 1.13, 95% CI: 1.02–1.24) for candidemia caused by other *Candida* species [16]. However, not all published cohorts have confirmed age as a consistent predictor of mortality in candidemia. For instance, the Spanish multicenter CANDIPOP study, which included non-*auris* candidemia, did not identify age as a predictor of early mortality in their multivariate model [17]. These contrasting data underscore the potential variability of prognostic factors depending on *Candida* species and even clades, supporting further investigation into the role of age in *C. auris* infection outcomes [12]. Our findings emphasize the need for heightened clinical vigilance in elderly patients, particularly in ICU settings.

In our cohort, previous corticosteroid use was independently associated with increased 30-day mortality (OR 4.06, 95% CI: 1.70–10.16). Although corticosteroid exposure has previously been recognized as a predisposing factor for fungal infections, including *C. auris*, and a recent meta-analysis found an association between previous corticosteroid use and unfavorable outcomes in candidemia overall (OR 2.27, 95% CI: 1.94–2.66) [18], it has not been identified as an independent predictor of mortality in *C. auris* candidemia [10]. A biologically plausible explanation relates to the immunosuppressive effects of corticosteroids, particularly their impact on neutrophil function and CD4+ lymphocyte counts, which may compromise host defenses against fungal invasion, thereby facilitating bloodstream dissemination, especially in critically ill patients [19]. However, this interpretation should be approached with caution, as the observed association may still be influenced by confounding factors not fully captured in the available data. In our cohort, an association was found between previous corticosteroid use and COVID-19, although no independent association between COVID-19 and mortality was observed. Moreover, corticosteroid exposure may also reflect greater baseline severity of illness or other underlying conditions that contributed to clinical outcomes.

Shock within 48 h of BSI/candidemia identification was the strongest predictor of mortality in our cohort (OR 6.22, 95% CI: 2.52–16.52). This is consistent with a prior study that identified shock as a major determinant of mortality in candidemia in a Colombian cohort that included *C. auris* and other species of *Candida* (aHR of 1.73, 95% CI: 1.41–2.13) [4]. The robust association observed between early shock and poor outcomes reinforces the importance of early hemodynamic deterioration as a clinical warning sign of poor prognosis, as well as the need for aggressive supportive care and prompt antifungal treatment.

The EQUAL Candida Score was inversely associated with mortality in our study (OR 0.84, 95% CI: 0.73–0.97). Patients with higher scores, reflecting closer alignment with recommended diagnostic and therapeutic interventions, had lower 30-day mortality. These findings support the potential utility of structured quality indicators in managing *C. auris* candidemia and are consistent with a recent study that applied the EQUAL Candida Score to *C. tropicalis* candidemia, in which higher scores were predictive of more favorable outcomes [14]. The variables included in this self-audit tool align with the recommendations included in international and local guidelines regarding the diagnosis, management, follow-up, and prevention of candidemia [20,21]. However, these observations should be interpreted in light of some limitations, particularly the inability to include all relevant variables due to missing data regarding the volume of blood cultures obtained, as well as the performance of ophthalmoscopy and echocardiography, and therefore the impact of these missing components on the prognostic performance of the score in *C. auris* candidemia remains uncertain. Notably, the unavailable variables accounted for 5 of the 22 total points of the EQUAL Candida Score. Thus, while the majority of score components were assessable, missing data may have affected the final scores, which should be considered when interpreting its association with mortality in our cohort. Nonetheless, no studies to date have evaluated the independent association between these variables and mortality. Additionally, survivorship bias should be considered, as patients who died early may have had less opportunity to undergo follow-up blood cultures, complete the recommended duration of antifungal treatment, or receive other components of guideline-based care. Consequently, lower EQUAL Candida Scores among patients with early mortality may partly reflect the limited time to complete these interventions rather than differences in quality of care alone.

From a clinical standpoint, the EQUAL Candida Score may help standardize clinical practice and promote adherence to guideline recommendations. However, its implementation in resource-limited healthcare settings may be challenging because some components, including routine echocardiography, ophthalmologic evaluation, and adherence to recommended blood culture volumes and follow-up blood cultures, may not be readily available, particularly in centers with restricted access to blood culture bottles. Nonetheless, the EQUAL Candida Score may remain valuable as a framework for prioritizing feasible interventions in candidemia management and identifying areas for improvement in antifungal stewardship.

Although research specifically focused on mortality in *C. auris* candidemia remains limited, our findings are partially consistent with previously published international and Colombian cohorts, although some differences warrant discussion. For instance, a recent multicenter retrospective cohort study from the United States, which included 187 patients, identified hemodialysis and a higher Pitt bacteremia score as independent predictors of mortality. In contrast, neither the Pitt score nor hemodialysis reached statistical significance in our multivariate model [22]. In that cohort, comorbidities and invasive medical devices, although common among non-survivors, were not independently associated with 30-day mortality, which is consistent with our findings. However, some comorbidities, such as diabetes mellitus and malignancy, have previously been reported as independent predictors of poor outcomes in a Colombian study of non-*C. auris* candidemia [16]. Meanwhile, another Colombian cohort focusing on *C. auris* candidemia found that the absence of antifungal treatment was associated with a higher risk of death (HR 5.52; 95% CI: 3.56–11.42), whereas delays in treatment initiation did not reach statistical significance [4]. Similarly, in our analysis, delayed antifungal treatment was not associated with increased 30-day mortality after adjustment. However, this finding should be interpreted cautiously, as the timing of antifungal initiation in clinical practice may be influenced by multiple factors, including prior antifungal exposure, disease severity, and diagnostic uncertainty, thereby complicating assessment of its relationship with mortality.

As mentioned previously, most available studies focus primarily on clinical and microbiological outcomes or compare prognosis between *C. auris* candidemia and candidemia caused by other *Candida* species rather than integrating prognostic factors with structured measures of quality of care. In this context, our study contributes data from a multicenter Latin American cohort and expands current evidence by evaluating the EQUAL Candida Score as a structured quality-of-care measure and potential prognostic marker in *C. auris* bloodstream infection.

As expected, hospital stay was longer among survivors than among patients who died. The prolonged hospital stay in this group may reflect the complex clinical context in which this infection occurs, with a median stay exceeding two months [23].

Antifungal susceptibility patterns vary considerably across geographic regions, largely reflecting the distribution of distinct phylogenetic clades [10]. Large multicenter susceptibility studies from India, where clade I predominates, have consistently reported fluconazole resistance rates of 90–100%, amphotericin B resistance rates ranging from 23% to 40%, and relatively low echinocandin resistance rates (2–7%). Approximately 40% of isolates were multidrug-resistant (MDR), whereas extensively drug-resistant (XDR) isolates remain uncommon (~4%) [11,15,24,25]. Beyond South Asia, phylogenetic analyses have demonstrated multiple introductions of distinct *Candida auris* clades into Europe, North America, and Latin America, likely contributing to the marked geographic variability in antifungal susceptibility [1,11]. In Latin America, although the South American clade (clade IV) predominates in Colombia and Venezuela, clades I and III have also been identified, particularly in Brazil and Argentina. Consequently, antifungal susceptibility varies substantially across the region. Colombian studies have generally reported lower fluconazole resistance rates (12.5–37%) than several other Latin American series (53–77%, reaching up to 100% in some reports) while maintaining high susceptibility to echinocandins; amphotericin B resistance has ranged from 21% to 75% [3].

In our cohort, fluconazole resistance reached 74.5%, substantially higher than previously reported in Colombian series (18% in 2019 and 37% in 2024), whereas echinocandin susceptibility remained preserved [3]. Although direct comparisons should be interpreted cautiously because of potential differences in study populations and susceptibility testing methods, this finding may suggest an increasing trend in fluconazole resistance over time. Continued national surveillance is needed to determine whether this reflects a true epidemiologic shift in circulating *C. auris* isolates.

This study has several limitations. First, its retrospective nature introduces the possibility of information bias and limits the ability to establish causal relationships. Second, microbiological identification was not performed using a uniform approach across all participating centers, and confirmation using reference methods was available only for a subset of isolates referred to the Instituto Nacional de Salud (INS). In addition, some isolates were initially identified by local methods as *C. auris/haemulonii* or *C. haemulonii*, which may have introduced misclassification despite the high confirmation rate among isolates undergoing reference testing. Third, antifungal susceptibility testing was performed in only for a proportion of isolates (38%), and amphotericin B susceptibility was not assessed. This limited our ability to fully characterize antifungal resistance patterns and explore potential associations between *in vitro* susceptibility, antifungal selection, and clinical outcomes, despite the clinical relevance of resistance patterns in candidemia management. Furthermore, because susceptibility testing was not systematically available across all participating centers, the resistance profile observed in this subset may not fully represent that of the entire cohort. Because this is a multicenter study, important differences in antifungal use were observed, which may have contributed to the difficulty in assessing relationships between treatment, susceptibility, and outcomes. More standardized antifungal practices should be incorporated into clinical protocols to improve understanding of these relationships. Finally, we only consider one episode per patient, since it is very difficult to determine whether more than one candidemia event might have occurred in the same hospitalization.

Moreover, the EQUAL Candida Score could not be fully assessed in all patients due to missing data on blood culture volume, ophthalmoscopy, and echocardiography, which together account for 5 of the 22 total points of the score. Although most score components were available, incomplete evaluation of the variables may have influenced the final score and its interpretation. Notably, these evaluations are not routinely performed in many hospitals across the region, which may have contributed to their incomplete availability in this cohort.

Nonetheless, this study has important strengths, including being one of the most extensive clinical descriptions of *C. auris* BSI in Colombia and including data from multiple high-complexity centers. Additionally, the EQUAL Candida Score was assessed for the first time as a quality-of-care metric for this pathogen, representing a novel approach that may inform quality improvement strategies in antifungal stewardship.

## 4. Materials and Methods

### 4.1. Study Design and Population

A multicenter retrospective cohort study was conducted. The inclusion criteria were adult patients (aged ≥18 years) with bloodstream infection, documented by at least one positive sample for *C. auris* from seven hospitals in Bogotá and Valledupar, Colombia. A previous evaluation of this cohort has been published [4]. WHONET 5.6 software (WHO, Collaborating Centre for Surveillance of Antimicrobial Resistance, Boston, MA, USA), containing microbiological information from the participating centers, was used to identify included patients between 2016 and 2021. The medical records of each identified patient were reviewed, and the information of interest (sociodemographic, clinical, and paraclinical variables) was recorded in an online data collection form, REDCap ver 14.1.14 (Vanderbilt University, Nashville, TN, USA). Identified patients were included regardless of whether antifungal treatment had been initiated. Exclusion criteria included duplicate patients, patients with no available electronic medical record, and identification of yeasts other than *C. auris*.

Participating centers in Bogotá included the Hospital Universitario Nacional de Colombia (HUN); Hospital Simón Bolívar of the Subred Integrada de Servicios de Salud Norte E.S.E. (HSB); Hospital de Kennedy of the Subred Integrada de Servicios de Salud Suroccidente E.S.E. (HOK); and Hospital El Tunal of the Subred Integrada de Servicios de Salud Sur E.S.E (HET). Participating centers in Valledupar included the Clínica Integral de Emergencias Laura Daniela (CELD); Instituto Cardiovascular del Cesar (ICVC); and Clínica Alta Complejidad del Caribe (CACC).

### 4.2. Microbiological Information

Microbiological data were extracted from each laboratory. Data were collected between 2016 and 2021 according to the protocols of each center, using the available microbiological identification methods. A proportion of *C. auris* isolates were confirmed at the Microbiology Laboratory of the National Institute of Health in Colombia (Instituto Nacional de Salud (INS)) using an MALDI-TOF MS Biotyper (Bruker Daltonics, Billerica, MA, USA), MBT version 4.1.80 and/or PCR [26,27]. A sample of isolates identified in Valledupar was confirmed by a single-tube PCR method based on amplification of internal transcribed spacer (ITS) regions, as previously described [28]. In Bogotá, one participating center has used MALDI-TOF MS technology as a microbiological identification method since 2017 (HOK), and the other three centers use phenotypic identification methods, such as MicroScan Walkaway^®^ (HET) and BD Phoenix^®^ (HET, HUN, and HSB). Isolates of *C. auris* included samples initially identified as *C. auris*, *C. auris/haemulonii*, and *C. haemulonii* [29]. Of the isolates included in this study as *C. auris*, 58 were sent to the INS, and 56 (96.6%) were confirmed as *C. auris,* whereas 2 were discarded because they were identified as other *Candida* species and not included in this study. Three patients were identified in the first blood culture as having non-*C. auris Candida* species (*C. albicans*, *C. utilis*, and *C. parapsilosis*); however, *C. auris/haemulonii* was identified in follow-up blood cultures. These cases were considered mixed candidemia and were included in this study. Susceptibility testing was performed using Clinical & Laboratory Standards Institute (CLSI) resistance breakpoints [30] as follows: fluconazole ≥ 32 µg/mL and caspofungin ≥ 2 µg/mL. No susceptibility testing was performed for amphotericin B.

### 4.3. Covariables

The date of blood culture collection was defined as day 0. Appropriate treatment was defined as the initiation of a susceptible antifungal agent between days 0 and +2, whereas late treatment was defined as initiation after day +3. ICU stay was considered when candidemia occurred within 14 days before or after ICU admission. Other variables studied included serum creatinine; the ratio between the fraction of inspired oxygen and arterial oxygen pressure; the fraction of inspired oxygen; organ dysfunction; sepsis; dialysis support; parenteral nutrition; Sequential Organ Failure Assessment (SOFA) score for sepsis; use of vasoactive medications; mechanical ventilation prior to isolation; previous surgery (within 3 months); previous antifungal use; previous antibiotic use; previous bacteremia; time to candidemia (from admission); Charlson comorbidity score; and the presence of cancer, diabetes mellitus, chronic kidney disease (CKD), chronic obstructive pulmonary disease (COPD), cardiovascular disease, and COVID-19. Previous corticosteroid use was considered for any dose administered before the index date of the candidemia. Age, gender, and the two hospitals that contributed the largest number of patients (HET and HSB) were also included. The EQUAL Candida Score was calculated for all patients, incorporating key management components such as performance of antifungal susceptibility testing, use of an echinocandin as initial therapy with de-escalation to fluconazole when appropriate, duration of antifungal treatment, and CVC removal. Data on blood culture volume, routine echocardiography, and ophthalmological evaluations were unavailable, and zero points were added for the missing information or if these procedures had not been routinely done. Shock was defined as the need for drugs to maintain blood pressure or a serum lactate concentration > 2 mmol/L. Shock was also assessed at 48 h as a marker of early deterioration.

### 4.4. Outcomes and Statistical Analysis

Descriptive statistics were performed using relative and absolute frequencies. Variables are described using means, standard deviations, medians, and interquartile ranges (25th–75th percentiles), according to the normal distribution. For some variables with less than 5% missing data, imputation was performed using mean or expected values according to the clinical context.

The outcome (dependent) variable was death at 30 days. A multivariate logistic regression model was used, and independent variables were selected based on bivariate analysis with *p* < 0.2 and relevant clinical data. The model with the lowest error and the greatest amount of information possible was selected after evaluating collinearity, confounding, and interactions. Goodness of fit was evaluated using the Hosmer–Lemeshow test. Discrimination of the model was estimated using the AUC-ROC. All statistical calculations were performed using R (ver. 4.6.1; The R Foundation for Statistical Computing, Vienna, Austria). A *p*-value of 0.05 was used for statistical significance.

## 5. Conclusions

Our findings add to the growing body of evidence on prognostic factors in *C. auris* BSI, identifying older age, prior corticosteroid exposure, and early shock development as strong predictors of 30-day mortality. Importantly, greater adherence to recommended candidemia management practices, as measured by the EQUAL Candida Score, may be associated with lower mortality risk. These results emphasize the importance of structured, evidence-based care and prompt recognition of clinical deterioration to improve outcomes in patients with *C. auris* BSI.

## Figures and Tables

**Table 1:** Clinical and demographic characteristics of survivors and deceased patients from seven tertiary care hospitals in Colombia, 2016–2021.

Characteristics	All Patients (n = 134)	Survivors (n = 83)	Non-Survivors (n = 51)	*p*-Value
Male sex, no. (%)	86 (64.2)	55 (66.3)	31 (60.8)	0.648
Age in years, mean (SD)	54.2 (17.1)	51.06 (17.2)	59.4 (15.7)	0.005
Comorbidities, no. (%)				
COVID-19 disease	47 (35.1)	24 (28.9)	23 (45.1)	0.086
Cardiovascular disease	44 (32.8)	23 (27.7)	21 (41.2)	0.155
Diabetes mellitus	30 (22.4)	18 (21.7)	12 (23.5)	0.972
Previous surgery	24 (17.9)	17 (20.5)	7 (13.7)	0.448
Chronic kidney disease	14 (10.4)	10 (12.0)	4 (7.8)	0.630
Solid tumor	14 (10.4)	10 (12.0)	4 (7.8)	0.630
Cancer	13 (9.7)	7 (8.4)	6 (11.8)	0.740
Chronic obstructive pulmonary disease	12 (9.0)	6 (7.2)	6 (11.8)	0.561
Chronic heart failure	7 (5.2)	1 (1.2)	6 (11.8)	0.023
Acute myocardial infarction	6 (4.5)	4 (4.8)	2 (3.9)	1.00
Peripheral vascular disease	4 (3.0)	2 (2.4)	2 (3.9)	1.00
Cerebrovascular disease	4 (3.0)	3 (3.6)	1 (2.0)	0.981
Dementia	1 (0.7)	0	1 (2.0)	0.805
Connective tissue disorders	1 (0.7)	0	1 (2.0)	0.805
Charlson score, mean (SD)	2.35 (2.11)	2.16 (2.29)	2.67 (1.76)	0.176
Predisposing factors, no. (%)				
ICU admission	116 (86.6)	68 (81.9)	48 (94.1)	0.08
Previous healthcare-associated infection	31 (23.1)	18 (21.7)	13 (25.5)	0.767
Previous antifungal exposure	19 (14.2)	13 (15.7)	6 (11.8)	0.709
Use of immunosuppressants	7 (5.2%)	3 (3.6)	4 (7.8)	0.504
Previous corticosteroid use	40 (29.9)	16 (19.3)	24 (47.1)	0.001
Mechanical ventilation	83 (61.9)	47 (56.6)	36 (70.6)	0.152
Parenteral nutrition	33 (24.6)	24 (28.9)	9 (17.6)	0.206
Renal replacement therapy	18 (13.4)	11 (13.3)	7 (13.7)	1.00
Previous bacteremia, no. (%)	40 (29.9)	25 (30.1)	15 (29.4)	1.00
Shock, no. (%)	32 (23.88)	15 (18.0)	17 (33.3)	0.071
SOFA score, median (SD)	7.4 (3.3)	6.7 (31)	8.6 (3.3)	0.001
Shock within 48 h of BSI, no. (%)	37 (27.6)	14 (16.9)	23 (45.1)	0.001
Pitt bacteremia score, mean (SD)	2.42 (1.94)	2.06 (1.74)	3.0 (2.12)	0.006

**Table 2:** Treatment variables and outcomes of patients from seven tertiary care hospitals in Colombia, 2016–2021.

Variable	All Patients (n = 134)	Survivors (n = 83)	Non-Survivors (n = 51)	*p*-Value
EQUAL Candida Score (median (IQR))	11 (10–14)	13 (10–15)	10 (8–13)	<0.001
Species identification	134 (100)	83 (100)	51 (100)	1.0
Echinocandin use, no. (%)	88 (65.7)	56 (67.5)	32 (62.7)	0.710
Fluconazole use, no. (%)	34 (25.4)	22 (26.5)	12 (23.5)	0.857
Antifungal treatment for 14 days (median (IQR))	38 (28.4)	29 (34.9)	9 (17.7)	0.050
Catheter removal, no. (%)	86 (64.2)	55 (66.3)	31 (60.8)	0.648
Follow-up blood culture, no. (%)	83 (61.9)	59 (71.1)	24 (47.1)	0.009
Antifungal susceptibility testing	51 (38)	----	---	---
Received antifungal treatment, no. (%)	119 (88.8)	74 (89.2)	45 (88.2)	1.00
Antifungal initiation within the first 48 h after diagnosis, no. (%)	48 (35.8)	24 (28.9)	24 (47.1)	0.052
Late antifungal initiation, no. (%)	71 (52.2)	50 (60.2)	21 (41.17)	0.057
Hospital stay, days (median (IQR))	51.5 (29–73)	64 (45–80)	31 (19–45)	<0.001

**Table 3:** Multivariate logistic regression analysis of factors associated with 30-day mortality in patients from seven tertiary care hospitals in Colombia, 2016–2021.

Variable	Odds Ratio	95% Confidence Interval
EQUAL Candida Score	0.84	0.73–0.97
Age (per decade)	1.37	1.07–1.78
Previous corticosteroid use	4.06	1.70–10.16
Shock within 48 h of BSI	6.22	2.52–16.52

## Data Availability

Anonymized data are available upon request.
